# Study on stability analysis of soil nail reinforced slopes under loading based on the discrete element method

**DOI:** 10.1371/journal.pone.0350163

**Published:** 2026-05-26

**Authors:** Fengling Tan, Guangjing Yin, Pengpeng Zhao, Guocheng Sun, Zhe Li, Zongtang Zhang

**Affiliations:** 1 Research and Design Institute, Sinohydro Engineering Bureau 8 CO., LTD., Changsha, Hunan, China; 2 Xiangyuan Zhenxing Information Technology Service Co., Ltd, Changsha, Hunan, China; 3 Hunan University of Science and Technology, Xiangtan, Hunan, China; China Construction Fourth Engineering Division Corp. Ltd, CHINA

## Abstract

Soil nailing is widely used to improve slope stability, yet the mechanical response of soil-nailed slopes subjected to surcharge loading remains insufficiently understood, particularly at the mesoscopic level. In this study, the discrete element method (DEM) was employed to investigate the progressive failure process, macroscopic stability, and micromechanical behaviors of a soil-nailed slope subjected to point loading and distributed linear loading with varying magnitudes and loading widths. The numerical results show that surcharge loading significantly reduces slope stability and increases deformation. Under point loading, the safety factor decreases continuously with increasing load magnitude and the deformation remains highly localized. Under linear loading, the safety factor decreases sharply as the loading width increases from 0 to approximately 1.5–2.0 m, and then tends to stabilize, whereas displacement continues to increase monotonically. Micromechanical analyses indicate that surcharge loading promotes rapid accumulation of elastic strain energy, intensifies particle rotation, and increases stress heterogeneity within the slope. Load distribution also strongly affects the force transfer mechanism in the reinforcement system: narrow loads are mainly resisted by upper nails, while wide distributed loads mobilize deeper nails and shift the stabilizing effect to lower reinforcement layers. The results suggest that surcharge loads near the slope crest should be carefully controlled, and that sufficient setback distance is important for limiting stress transmission into the active failure zone. These findings provide insight into the failure mechanism of soil-nailed slopes under surcharge loading and offer practical guidance for reinforcement design.

## 1. Introduction

Surcharge loading is a common external disturbance in slope engineering and may accelerate the development of plastic zones and slip surfaces within natural or excavated slopes, thereby degrading stability [1,2]. Soil nailing is a widely adopted reinforcement technique for slope stabilization because of its relatively simple construction, rapid installation, and favorable cost-effectiveness [3,4]. By inserting steel nails into the slope body and grouting them to improve soil-reinforcement interaction, soil nailing can effectively restrain lateral deformation, delay slip-surface propagation, and enhance overall slope resistance to failure [5,6]. However, the mechanical response of soil-nailed slopes under surcharge loading is highly complex, especially when the load is applied near the slope crest or distributed over a broad area.

A substantial body of research has examined slope stability under external loading [7,8], excavation, rainfall, and seismic disturbance [9,10]. Analytical approaches, numerical simulations, and strength reduction methods have been used to evaluate the influence of reinforcement layout, loading conditions, and soil parameters on slope stability [11,12]. These studies have improved the understanding of global stability behavior and have demonstrated that surcharge loading can induce stress concentration [13], plastic zone expansion, and excessive displacement near the slope crest [14]. In parallel, a number of studies have investigated the performance of soil-nailed slopes, focusing on the effects of nail length, inclination, spacing, and installation position on the safety factor and deformation response [5]. Nevertheless, most existing studies are based on continuum mechanics frameworks, which are well suited for macroscopic analysis but less capable of capturing particle-scale interactions, force-chain evolution, and progressive failure processes.

The discrete element method (DEM) offers a useful alternative for studying granular materials and geotechnical structures because it can explicitly simulate contact interactions, localized rearrangement [15,16], and discontinuous failure mechanisms [17–19]. Recent DEM-based studies have shown that this method can effectively capture force-chain development [20,21], strain localization [22–24], and internal load transfer in reinforced soil systems and slope structures [25- 26]. Compared with continuum methods, DEM is particularly advantageous for revealing the mesoscopic mechanisms that link external loading conditions to macroscopic deformation and failure [27,28]. Despite this advantage, relatively few studies have used DEM to investigate the surcharge response of soil-nailed slopes, especially from the perspective of load distribution, stress heterogeneity, and reinforcement force mobilization.

In addition, the effect of the spatial extent of surcharge loading near the slope crest remains insufficiently clarified. In practical engineering conditions, surcharge loads may be applied as concentrated point loads or as distributed loads with different widths, and these loading patterns may lead to different stress transmission paths and reinforcement responses. A clearer understanding of how load magnitude and load width influence the progressive failure mechanism of soil-nailed slopes is therefore needed. To address this gap, the present study uses DEM to analyze a soil-nailed slope subjected to point loading and distributed linear loading. The study systematically examines the evolution of safety factor, deformation, elastic strain energy, particle rotation, stress heterogeneity, and nail tensile force under different loading conditions. The objective is to clarify the macroscopic and micromechanical mechanisms governing slope response and to provide a theoretical basis for the design of soil-nailed slopes under surcharge loading.

## 2. Discrete element modeling

The discrete element method (DEM) is capable of simulating various constitutive behaviors of geotechnical materials, including plastic deformation, flow, viscoelasticity, viscoplasticity, creep, and two-phase interactions. It effectively captures large deformation and failure mechanisms in geomaterials [29]. In this study, PFC5.0, a famous DEM software, was employed to analyze the stability of soil nail reinforced slopes under different loading configurations.

### 2.1. Contact model

**Fig 1:**
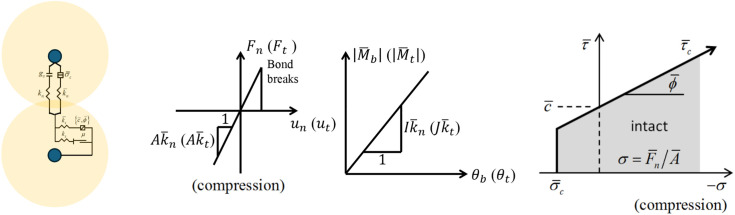
Notation used to describe soil-soil contacts.

In DEM simulations, particles are assumed as rigid balls, and deformation is controlled by overlapping based on contact force Contacts are deformable, allowing overlap, and the overlap depends on the contact force and force–displacement relationship. Particle contacts are bondable and can generate cohesive interactions. The typical linear parallel bond model is used to simulate the interaction of soil particles (Fig 1).

The force-displacement relationship can be expressed as (Zhang et al., 2021):


$$\begin{array}{c} F_{n}=\left\{ \begin{array}{c} \overset{―}{A}{\overset{―}{k}}_{n}u_{n}\\ \overset{―}{A}({\overset{―}{k}}_{n}+k_{n})u_{n} \end{array}\right.\; \end{array}$$ (1)

$$\begin{array}{c} F_{t}=\left\{ \begin{array}{c} \overset{―}{A}{\overset{―}{k}}_{t}u_{t}\\ \overset{―}{A}({\overset{―}{k}}_{t}+k_{t})u_{t} \end{array}\right.\; \end{array}$$ (2)

where $k_{n}$ and $k_{t}$ are the linear normal stiffness and the linear tangential stiffness. ${\overset{―}{k}}_{n}$ and ${\overset{―}{k}}_{t}$ are the parallel bond normal stiffness and the parallel bond tangential stiffness, respectively. $\overset{―}{A}$ is the contact area between bonded particles. Moreover, the bending moment $M_{b}$ and the twisting moment $M_{t}$ are defined as:


$$\begin{array}{c} M_{b}=\text{0}.\text{2}\text{5}\pi {\overset{―}{R}}^{\text{4}}{\overset{―}{k}}_{n}\theta_{b} \end{array}$$ (3)

$$\begin{array}{c} M_{t}=\text{0}.\text{5}\pi {\overset{―}{R}}^{\text{4}}{\overset{―}{k}}_{t}\theta_{t} \end{array}$$ (4)

where $\theta_{b}$ and $\theta_{t}$ are bend-rotation and twist-rotation, respectively.

It should be noted that several simplifying assumptions were adopted in the present DEM modeling framework. First, soil particles were treated as rigid bodies, and particle deformation was not explicitly considered. This assumption may influence the absolute magnitude of displacement; however, it is commonly adopted in DEM studies and is generally acceptable for capturing relative deformation trends and failure mechanisms. Second, the particle size and two-dimensional representation may introduce scale effects, which can affect stress diffusion and displacement localization. Despite these limitations, the adopted DEM approach is effective in revealing the mesoscopic interaction mechanisms and comparative responses of soil nail reinforced slopes under different surcharge loading patterns. Future studies will focus on incorporating deformable particles, advanced interface models, and three-dimensional simulations to further improve model fidelity.

### 2.2. Calibration of model parameters

**Fig 2:**
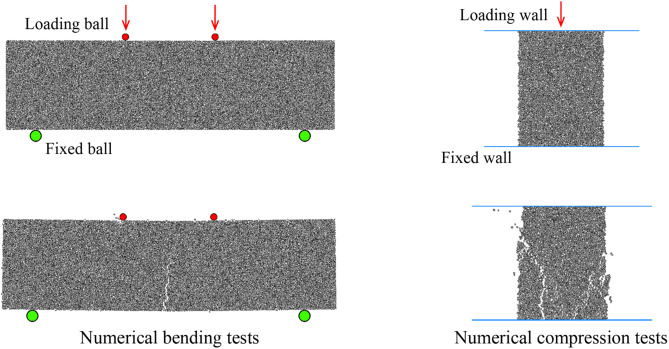
Numerical bending and compression tests.

The accuracy of DEM simulation depends on model parameters. In this study, the numerical simulation involves two materials: soil and concrete (nails). Therefore, we calibrated both the bending and compressive strengths of these two materials (Fig 2). According to previous studies [14], the contact properties between the two materials are assumed to be weaker, for example, the concrete-soil contact properties are equivalent to those of soil. In the bending test, balls are used to apply load, with the two upper balls pressing down at a constant strain ratio while the two lower balls remain fixed; in the compression test, walls are used to apply load. The particle size of the material in the test is consistent with the simulation (uniformly distributed with a radius of 0.05–0.075 m), and the loading strain rate is consistent with the real test (0.001/s).

In the calibration process, it is assumed that the friction coefficient under bonding conditions is consistent with that after bonding failure. The ratio of normal to tangential stiffness remains consistent under both bonded and unbonded conditions, set at 1.2 [30,31]. Ultimately, the bending strength of concrete is 4.4 MPa, and its compression strength is 27 MPa. The bending and compression strengths of soil are 2.7 kPa and 100 kPa, respectively. The model parameters are listed in Table 1.

**Table 1:** Model parameters.

Parameter	Value
Soil density (kg/m^3^)	2200
Nail density (kg/m^3^)	2500
Initial porosity	0.15
Friction coefficient of soil	0.18
Friction coefficient of nail	0.5
Linear and bond normal stiffness of nail (N/m)	1.2e10
Linear and bond normal stiffness of soil (N/m)	1e7
Stiffness ratio	1.2
Tensile strength of soil (MPa)	0.5
Tensile strength of nail (MPa)	14
Cohesion of soil, *c* (MPa)	0.5
Cohesion of nail (MPa)	200
Global damping	0.5

While the proposed DEM model effectively captures the macroscopic deformation and microscopic stress evolution of the soil-nail reinforced slope, certain limitations should be acknowledged. First, the soil particles in the current DEM framework are assumed to be perfectly rigid. Consequently, potential particle crushing or breakage, which might occur under severe localized stress concentrations (e.g., at the slope toe or directly beneath heavy point loads), is not accounted for. Second, the soil-nail interface is simulated using a simplified parallel bond model. Although this approach reasonably simulates the transfer of tensile and shear forces, it may not fully capture the complex, non-linear pull-out behaviors, such as interface dilation or the degradation of the cement grout in real-world scenarios. Despite these simplifications, the comparative trends regarding safety factors and failure mechanisms under varying surcharge loads remain highly reliable. Future work will aim to incorporate crushable particle models and more advanced interface constitutive laws to further improve model fidelity.

### 2.3. Slope modeling

**Fig 3:**
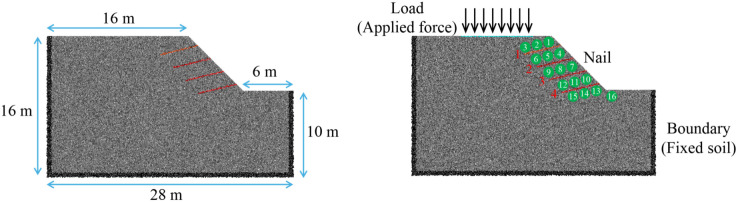
Geometry of the soil nail reinforced slope.

Due to site constraints in a highway project, materials were stockpiled at the crest of a temporary soil slope with a height of 6 m and a gradient of 1:1. A building was located near the slope toe, necessitating reinforcement with soil nails. According to the Technical Guidelines for Soil Nail Support of Highway Slopes (China Communications Press, 2006), the ratio of soil nail length _*L*_ to the supported slope height _*H*_ is generally recommended to fall within the range of 0.8–1.2 to ensure sufficient anchorage beyond the potential slip surface. For the investigated slope with a height of 6 m, a soil nail length of 6.0 m (i.e., *L/H* = 1.0) was adopted. Moreover, the angle between the nail and the horizontal plane is 15°. This value not only satisfies the code-recommended range but also represents a commonly used design choice in engineering practice for temporary soil nail reinforced slopes, providing an effective balance between reinforcement performance and construction feasibility. To evaluate slope stability under loading, a discrete element model was established. The model dimensions were 28 m in length, 16 m in height, with a slope angle of 45° and a crest length of 16 m. To gain a deeper understanding of the impact of reinforcement on slopes, we have set up 16 measuring circles in the reinforcement area of the numerical slope (Fig 3).

In simulation process, a layered compaction method was adopted for sample preparation, and a total of 24824 particles were generated. To simulate in situ confining pressure, a constant displacement boundary was applied at the model base, while servo-controlled boundaries were used on the sides. The servo system ensured that boundary stresses matched actual ground stress conditions:


$$\begin{array}{c} \sigma_{\text{xx}}=\sigma_{yy}=k_{\text{0}}\sigma_{zz} \end{array}$$ (5)

$$\begin{array}{c} k_{\text{0}}=\text{1}-sin\phi \end{array}$$ (6)

Here, *σ*_*x*_, *σ*_*yy*_ and *σ*_*zz*_ are the horizontal stresses in the direction of x, y and z respectively; k_0_ is the static earth pressure coefficient; ϕ is the internal friction angle of the soil. Assign zero velocity to all model elements when the ratio of maximum unbalanced force to total unbalanced force falling below 10^−5^ [27] Then, the particles at the edges were fixed to form a flexible boundary (set the velocity of particles on the left and right boundaries in the x direction to 0, and the velocity of particles at the bottom in the y direction to 0), while the particles at the nail positions were filtered out and assigned concrete parameters. The properties of nail-soil contacts are the same as the properties of soil-soil contacts.

In loading process, the load was directly applied on the soil particles to ensure no stress concentration occurs. There were two ways of loading: point loading and linear loading. In point loading, force was used as the unit, and all forces were applied to a single ball; in linear loading, the force was converted into uniformly distributed stress for application. Both point loading and line loading were applied starting from a position 2m away from the slope surface to simulate large-scale surcharge applied at the slope crest. A total of 26 conditions were designed (Table 2).

**Table 2:** Numerical test scheme.

loading type	Load magnitude	Length of force application (m)
Point	5, 10, 20, 40, 60 kN	–
Linear	50, 100, 200 kPa	0.5
		1.0
		1.5
		2
		4
		6
		8

## 3. Analysis of numerical results

In this section, a comprehensive macroscopic and micromechanical analysis is conducted based on the DEM simulation results. The mechanical response of the soil nail reinforced slope is evaluated under both concentrated point loads and distributed linear loads with varying magnitudes and loading lengths. By tracking macroscopic indicators (such as the factor of safety and maximum displacement) alongside micromechanical parameters (including particle stress, Gini coefficient, elastic strain energy, particle rotation, and internal forces within the soil nails), the complex progressive failure mechanisms and load transfer behaviors within the reinforced soil mass are systematically elucidated.

### 3.1. Macroscopic stability and deformation response

#### 3.1.1. Evolution of the safety factor.

Stability analyses were performed after equilibrium was reached under each loading condition [32]. The safety factor *F*_*s*_, the most direct indicator of slope stability, is obtained using the mesoscopic parameter reduction method, and the reduction factor at the time of slope failure is considered as the safety factor:


$$\begin{array}{c} \phi_{\text{failure}}={\text{tan}}^{-\text{1}}\left(\frac{\text{tan}\phi }{F_{s}}\right) \end{array}$$ (7)

$$\begin{array}{c} c_{\text{failure}}=\frac{c}{F_{s}} \end{array}$$ (8)

Using the trial and error method for calculation, the safety factor was accurate to two decimal places [9], with an average of over 30 trial calculations per model. The influence of different loading conditions on the reinforcement effectiveness of soil nails was thereby quantified.

**Fig 4:**
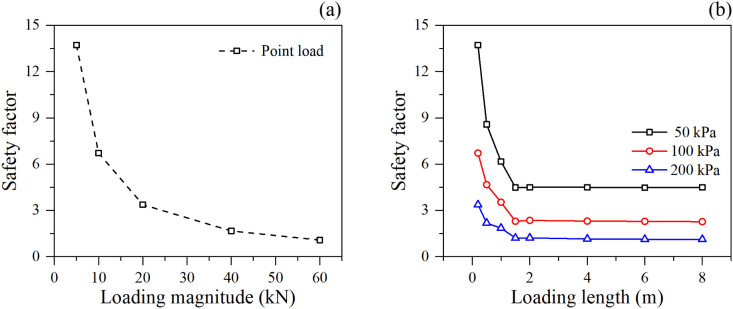
Safety factor under different loading condition: (a) point loading, and (b) linear loading.

As illustrated in the relationship between loading conditions and safety factor, the slope exhibits distinctly different stability deterioration modes under point loading and distributed linear loading. Under point loading conditions (Fig 4a), the safety factor demonstrates a continuous, non-linear decaying trend as the loading magnitude increases from 5 kN to 60 kN. The initially high safety margin rapidly depletes, approaching critical instability (safety factor nearing 1.0) when the load exceeds 40–50 kN. This indicates that concentrated loads induce severe local shear stress concentrations that effectively penetrate the reinforced zone.

Conversely, under distributed linear loading (Fig 4b), the safety factor exhibits a striking “sharp drop followed by a stable plateau” characteristic with respect to the loading length. For all applied surface pressures (50 kPa, 100 kPa, and 200 kPa), the safety factor decreases precipitously when the loading length expands from 0 m to approximately 1.5 m – 2.0 m. However, once the loading length exceeds this critical threshold (around 2.0 m), the safety factor ceases to decline and maintains a constant plateau, despite the total applied load continuously increasing with the loading length. This phenomenon can be fundamentally explained by the limit equilibrium theory and the spatial geometry of the potential active failure wedge. When the loading length is small, the load acts directly on the crest portion closest to the slope face, significantly increasing the driving forces acting on the critical slip surface. As the loading length extends backward, it eventually covers the entirety of the potential active wedge. Any further extension of the load beyond the rear boundary of the active wedge acts upon the stable soil mass (passive or stable zone) behind the slip surface. Consequently, this additional load does not contribute to the driving overturning moment or sliding force of the active wedge, resulting in a constant safety factor. Furthermore, higher loading magnitudes (e.g., 200 kPa) lower the overall horizontal plateau of the safety factor, with the 200 kPa load reducing the safety factor to near 1.0, highlighting an impending global failure [33].

#### 3.1.2. Maximum displacement of soil particles.

**Fig 5:**
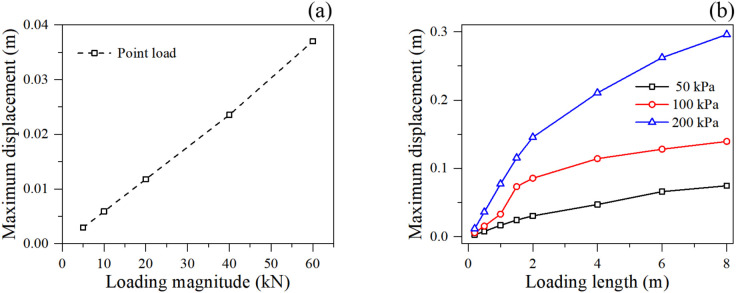
Maximum particle displacement under different loading condition: (a) point loading, and (b) linear loading.

The displacement response provides further insight into the slope’s kinematic behavior prior to failure. Under point loading (Fig 5a), the maximum displacement increases almost linearly with the loading magnitude, suggesting a localized punching shear mechanism where the surrounding soil provides a relatively constant stiffness against the concentrated force.

However, under linear loading (Fig 5b), the maximum displacement exhibits a non-linear, monotonically increasing trend with the loading length, which contrasts sharply with the plateauing behavior of the safety factor. As the loading length increases up to 8 m, the maximum displacement continues to grow significantly, particularly under the extreme load of 200 kPa, where the displacement reaches nearly 0.3 m. This continuous increase in displacement, even when the safety factor has stabilized, indicates that while the global catastrophic sliding plane might be fixed by the geometric boundaries of the active wedge, the soil mass is undergoing continuous volumetric compression, local yielding, and internal deformation under the massive surcharge. The larger the loaded area, the deeper the stress bulb penetrates, causing cumulative settlement and lateral bulging of the slope face.

### 3.2. Micromechanical behaviors

The unique advantage of DEM allows for the direct observation of internal micromechanical mechanisms that drive macroscopic deformation.

#### 3.2.1. Energy accumulation.

**Fig 6:**
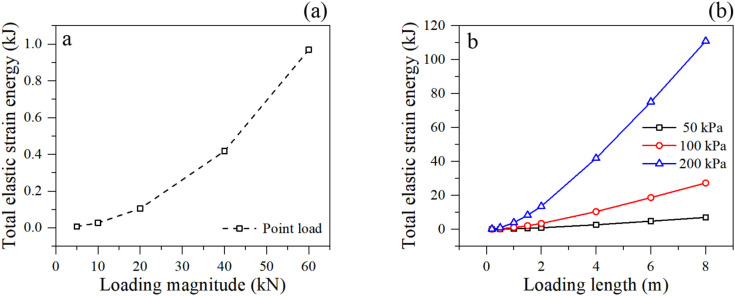
Elastic strain energy accumulation under different loading condition: (a) point loading, and (b) linear loading.

The total elastic strain energy within the granular system serves as a crucial barometer for the structural integrity of the slope. Fig 6 shows the total elastic strain energy of soil slope in different loading condition. The results show that point loads induce an almost quadratic increase in elastic strain energy, reflecting the rapid storage of elastic forces within strong force chains near the loading point [34].

Under linear loading, the strain energy exhibits a strong dependence on the loading magnitude. At lower pressures (50 kPa and 100 kPa), the energy increases moderately and linearly with loading length. However, under the 200 kPa load, a dramatic, non-linear surge in elastic strain energy is observed as the loading length expands. This massive accumulation of energy signifies that a vast network of force chains is being heavily mobilized to resist the external work done by the wide, heavy surcharge. In granular mechanics, such a sharp increase in stored elastic energy usually precedes a violent release phase (kinetic energy spike), which corresponds to the sudden rupture of force chains and the macroscopic collapse of the slope. The 200 kPa condition is thus identified as a critical state where the soil mass is fully “charged” and teetering on the edge of global plastic flow.

#### 3.2.2. Stress homogeneity.

In granular systems such as reinforced slopes, the transmission of macroscopic external loads is inherently heterogeneous. The load is typically carried by a sparse, self-organized network of heavily loaded contacts, known as strong force chains, while the majority of contacts transmit relatively weak forces. To quantitatively assess the degree of this contact force heterogeneity, the Gini coefficient (*G*), a statistical dispersion indicator fundamentally utilized in economics to represent wealth inequality, is introduced to evaluate the load distribution inequality among contacts. For a given measurement domain containing *N* active contacts, let the absolute values of the normal contact forces be sorted in ascending order, yielding a sequence $f_{1}\leq f_{2}\leq \ldots \ldots \leq f_{N}$. The Gini coefficient of the normal contact force network is mathematically defined as:


$$\begin{array}{c} G=\frac{2\sum_{k=1}^{N}kf_{k}}{N\sum_{k=1}^{N}f_{k}}-\frac{N+1}{N} \end{array}$$ (9)

where *k* denotes the rank index of the sorted contact force $f_{k}$. The value of the Gini coefficient *G* is strictly bounded between 0 and 1. A state of G=0 corresponds to perfect homogeneity, signifying that all inter-particle contacts carry identical normal forces. Conversely, as *G* approaches 1, it indicates extreme heterogeneity, implying that the macroscopic loads are intensely concentrated on a minimal fraction of contacts. During the slope failure process, an abrupt increase in *G* generally serves as a microscopic precursor to macroscopic instability, reflecting the buckling of strong force chains and the subsequent stress redistribution.

**Fig 7:**
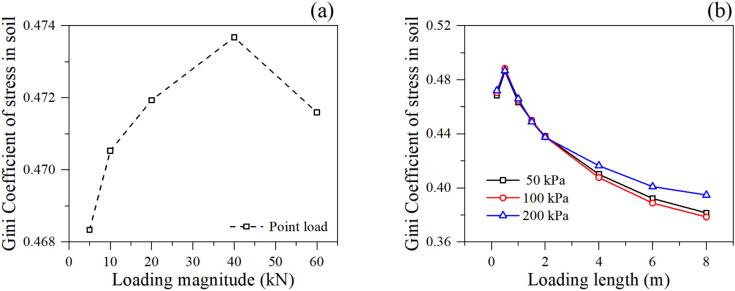
The Gini coefficient of soil particle stress under different loading condition: (a) point loading, and (b) linear loading.

The Gini coefficients of soil particle stress in different loading condition are shown in Fig 7. Interestingly, the Gini coefficient exhibits completely different trends under the two loading types. Under point loading (Fig 7a), the Gini coefficient initially increases with the load magnitude (from 5 to 40 kN), indicating severe stress concentration directly beneath the point of application. However, a drop is observed at 60 kN, suggesting that local yielding or failure has occurred beneath the point load, forcing the stress to redistribute to surrounding intact soil particles, thereby slightly reducing the extreme localization.

Under linear loading (Fig 7a), the Gini coefficient decreases monotonically as the loading length increases. This is because a wider load applies pressure over a broader area of the slope crest, effectively homogenizing the stress field across the upper soil mass. The load transitions from a localized stress concentrator to a uniform overburden pressure, reducing spatial stress disparities.

#### 3.2.3. Kinematic behavior of soil particles.

**Fig 8:**
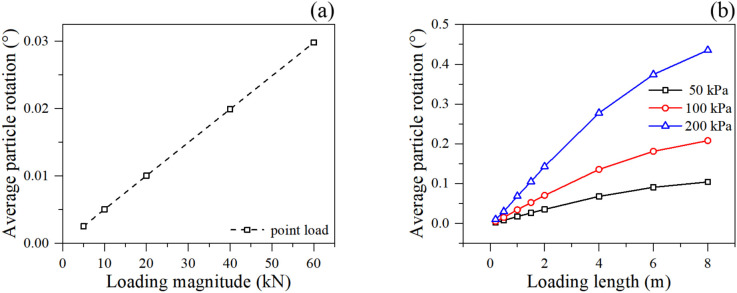
Rotation of soil particles under different loading condition: (a) point loading, and (b) linear loading.

The average particle rotation (shown in Fig 8) is a crucial indicator of shear band formation in granular materials, as particles tend to roll significantly within actively shearing zones. For the point load, the average particle rotation increases linearly with the load magnitude, indicating the progressive development of a localized shear zone propagating downwards from the loading point. For the linear load, the particle rotation increases substantially with both loading magnitude and length. Notably, the 200 kPa linear load induces a massive increase in particle rotation compared to 50 kPa. The continuous increase in rotation with loading length confirms that a wider load expands the volume of the active shear bands within the slope, forcing more soil particles into intense frictional rolling and sliding.

### 3.3. Load transfer and soil-nail interaction mechanisms

The core stabilizing mechanism of the reinforced slope lies in the mobilization of tension within the soil nails. The numerical results reveal highly complex load-sharing and stress-transfer dynamics among the multiple nail layers.

#### 3.3.1. Mobilization of tensile forces in nails.

**Fig 9:**
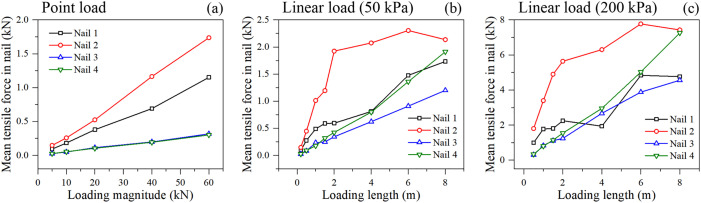
Tensile forces in nails under different loading condition: (a) point loading, (b) linear loading of 50 kPa, and (c) linear loading of 200 kPa.

The mean tensile force in the nails highlights the sequence of load mobilization. Tensile forces in nails under different loading condition are illustrated in Fig 9. Under point loading (Fig 9a), Nail 2 (located in the upper-middle section) consistently bears the highest tensile force, followed by Nail 1, while deeper nails (Nail 3 and 4) remain relatively unstressed. This indicates that the failure wedge induced by a concentrated load near the crest is relatively shallow and localized in the upper strata.

Under linear distributed loading (Fig 9), the load redistribution is highly dynamic and depends critically on the loading length. For smaller loading lengths (e.g., < 2 m), Nail 2 again dominates the tensile load carrying capacity, counteracting the shallow active wedge. However, as the loading length continues to extend backwards (up to 8 m), a profound shift occurs. While the tension in the upper nails (Nail 1 and 2) plateaus or even slightly decreases, the tensile forces in the deeper nails (Nail 3 and Nail 4) surge dramatically. This is most pronounced under the 200 kPa load, where Nail 4 (the deepest layer) eventually overtakes the upper nails to bear an enormous tensile load at large loading lengths.

This micro-mechanical evidence perfectly maps to a macro-mechanical shift in the failure mode. As the heavy surface load expands far behind the crest (Yang et al., 2024), the critical slip surface is pushed significantly deeper into the foundation. The shallow nails are no longer sufficient or optimally positioned to intercept this deep-seated failure plane; instead, the deep nails are heavily mobilized as the massive, deep-seated active wedge attempts to slide forward.

#### 3.3.2. Evolution of soil-nail contact forces.

**Fig 10:**
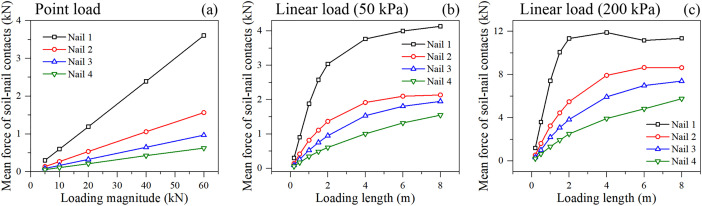
Mean force of soil-nail contacts under different loading condition: (a) point loading, (b) linear loading of 50 kPa, and (c) linear loading of 200 kPa.

The mean force of soil-nail contacts further corroborates this observation (Fig 10). The contact force measures the direct interaction (shear and normal stresses) between the granular soil particles and the nail interface. Under the 200 kPa linear load, the contact forces on Nail 1 quickly saturate and slightly decline after a loading length of 3 m. Simultaneously, the contact forces on Nail 3 and 4 rise continuously. This suggests that the soil around the uppermost nail may have fully yielded or softened (post-peak state), transferring the structural demand downwards to the intact soil-nail interfaces in the deeper layers.

**Fig 11:**
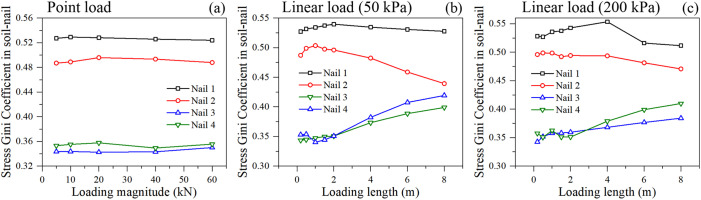
The stress Gini coefficient of soil-nail contacts under different loading condition: (a) point loading, (b) linear loading of 50 kPa, and (c) linear loading of 200 kPa.

Furthermore, the stress Gini coefficient within the soil-nail system (Fig 11) exhibits a diverging trend. For upper nails, the Gini coefficient decreases or stabilizes, indicating uniform stress along the nail as it fully engages. For lower nails, the Gini coefficient rises with loading length, indicating localized stress concentrations along the nail shaft as it begins to actively intercept the advancing deep shear band.

#### 3.3.3. Deep-seated stress propagation.

**Fig 12:**
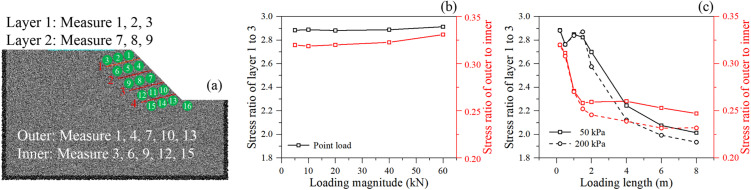
Stress ratio of different position of slope: (a) position of the measurement circle, (b) point loading, and (c) linear loading.

The data from the measurement circles embedded within the slope (Stress ratio of outer to inner layers) provide a similar evidence. The ratio of outer (near slope face) to inner (deep inside the slope) stress plummets as the linear loading length increases. A high initial ratio means stress is primarily confined to the slope face (Fig 12). The sharp decline indicates that widening the load effectively drives the stress bulb deep into the inner core of the slope. This confirms that long, distributed surface loads transform the failure mechanism from a shallow face-sloughing mode to a massive, deep-seated global rotational/translational sliding mode.

### 3.4. Discussion

The DEM numerical analysis reveals that loading conditions fundamentally alter the failure mechanics of soil nail reinforced slopes. While point loads trigger localized shear failures primarily resisted by upper nail layers, wide distributed loads induce deep-seated stress propagation. The initial widening of a surface load rapidly deteriorates the factor of safety until it fully covers the active wedge. However, further widening continues to cause massive energy accumulation, widespread particle rotation, and deep internal deformations. Crucially, wide surface loads force a radical redistribution of internal forces, shifting the stabilizing burden from the shallow nails to the deepest nail layers [35].

From an engineering perspective, these findings emphasize that when designing soil nail systems for areas subjected to extensive surface surcharges (such as large storage yards or multi-lane highways near the crest), the length and tensile capacity of the bottom-most nails must be significantly enhanced. Relying on uniform nail designs may lead to catastrophic deep-seated failures, as the lower nails will experience sudden and severe tensile force spikes once the surface load exceeds a critical spatial extent. Moreover, classical elastic theory suggests that surcharge loads applied near the slope crest generate a stress bulb that propagates downward and laterally into the slope body. When the loading area is located close to the crest, a substantial portion of this stress bulb overlaps with the potential active sliding zone, leading to higher shear stress concentration and larger horizontal deformation. As the loading width increases, the induced stress field extends deeper into the slope and gradually redistributes within the soil mass, which explains the observed reduction and eventual plateau in the safety factor once the loading width exceeds a critical range. Beyond this range, further expansion of the loading width has only a limited effect on the global stability index, although the internal deformation and energy accumulation continue to increase. These DEM results are consistent with the stress diffusion mechanism of surcharge loading and indicate that the spatial extent of the load, rather than its magnitude alone, plays a decisive role in governing the interaction between surcharge loading and the potential failure zone. These micro-mechanical findings theoretically validate the engineering consensus that surface surcharge setbacks are more effective than simply reinforcing superficial layers.

## 4. Conclusions

In this study, the discrete element method (DEM) was employed to systematically investigate the mechanical response, failure mechanisms, and soil-nail interactions of nail-reinforced slopes under varying loading conditions (point and linear loads). Based on the comprehensive macro-microscopic analysis, the following main conclusions can be drawn:

(1) Surcharge loading significantly degrades the stability of the soil-nailed slope. Under point loading, the safety factor decreases nonlinearly with increasing load magnitude, and the deformation remains strongly localized near the loading position. Under linear loading, the safety factor drops rapidly as the loading width increases from 0 to approximately 1.5–2.0 m, after which it tends to reach a plateau within the investigated range. This indicates that widening the load initially increases the driving effect on the active wedge, but further widening has a limited influence on the global safety factor once the active zone is fully covered.
(2) The macroscopic deformation and micromechanical evolution are consistent with progressive failure of the slope. Surcharge loading causes continuous accumulation of elastic strain energy, increases particle rotation, and intensifies stress heterogeneity within the soil mass. These responses indicate that the slope undergoes increasing internal rearrangement and localized shear development as loading becomes larger or more widely distributed.
(3) The load-transfer mechanism within the reinforcement system changes with loading pattern. Under point loading and narrow linear loading, the upper nails primarily resist the imposed load. As the loading width increases, deeper nails are increasingly mobilized, and the tensile force distribution shifts downward. This demonstrates that wide surcharge loading can drive the potential failure surface deeper into the slope body and increase the demand on lower reinforcement layers.
(4) The spatial position of surcharge loading is critical to slope performance. For the geometry and material parameters adopted in this study, the results suggest that surcharge placed close to the slope crest has a much stronger destabilizing effect than surcharge placed farther back. A setback distance of approximately 3.0 m appears sufficient to reduce the direct influence of surcharge on the active failure zone under the present modeling conditions. However, this value should be interpreted as case-specific and not as a universal design criterion. For practical engineering applications, the reinforcement design should be adjusted according to slope geometry, soil properties, and the expected range of surcharge loading.

## Supporting information

S1 File Data. (ZIP)
